# Antibacterial Activity of Clay Soils against Food-Borne *Salmonella typhimurium* and *Staphylococcus aureus*

**DOI:** 10.3390/molecules27010170

**Published:** 2021-12-28

**Authors:** Nur Naqiyah Azmi, Nor Ainy Mahyudin, Wan Hasyera Wan Omar, Nor-Khaizura Mahmud Ab Rashid, Che Fauziah Ishak, Abdul Halim Abdullah, Gary J. Sharples

**Affiliations:** 1 Halal Products Research Institute, Universiti Putra Malaysia, Serdang 43400, Malaysia; n.naqiyahazmi@gmail.com (N.N.A.); hasyera92@gmail.com (W.H.W.O.); 2 Faculty of Food Science and Technology, Universiti Putra Malaysia, Serdang 43400, Malaysia; norkhaizura@upm.edu.my; 3 Faculty of Agriculture, Universiti Putra Malaysia, Serdang 43400, Malaysia; cfauziah@upm.edu.my; 4 Faculty of Science, Universiti Putra Malaysia, Serdang 43400, Malaysia; halim@upm.edu.my; 5 Department of Biosciences, Durham University, Durham DH1 3LE, UK; gary.sharples@durham.ac.uk

**Keywords:** antibacterial clay, sedimentation, sieving, clay leachates, clay suspensions, *Salmonella typhimurium*, *Staphylococcus aureus*

## Abstract

Natural clays have recently been proven to possess antibacterial properties. Effective natural antimicrobial agents are needed to combat bacterial contamination on food contact surfaces, which are increasingly more prevalent in the food chain. This study sought to determine the antibacterial activity of clays against the food-borne pathogens *Salmonella typhimurium* ATCC 14028 and *Staphylococcus aureus* ATCC 13565. Soils were processed to yield leachates and suspensions from untreated and treated clays. Soil particle size, pH, cation-exchange capacity, metal composition and mineralogy were characterized. Antibacterial screening was performed on six Malaysian soils via the disc diffusion method. In addition, a time-kill assay was conducted on selected antibacterial clays after 6 h of exposure. The screening revealed that Munchong and Carey clays significantly inhibit *Salmonella typhimurium* (11.00 ± 0.71 mm) and *S. aureus* (7.63 ± 0.48 mm), respectively. Treated Carey clay leachate and suspension completely kill *Salmonella typhimurium*, while *S. aureus* viability is reduced (2 to 3 log_10_). The untreated Carey and all Munchong clays proved ineffective as antibacterials. XRD analysis confirmed the presence of pyrite and magnetite. Treated Carey clays had a higher soluble metal content compared to Munchong; namely Al (92.63 ± 2.18 mg/L), Fe (65.69 ± 3.09 mg/L) and Mg (88.48 ± 2.29 mg/L). Our results suggest that metal ion toxicity is responsible for the antibacterial activity of these clays.

## 1. Introduction

Clay has been employed as a natural remedy since prehistory. Aside from its healing and soothing properties, clay has been investigated for its antibacterial activities, exemplified by the successful application of French green clay in the treatment of Buruli ulcer, a necrotizing cutaneous infection caused by *Mycobacterium ulcerans* [1] and the antibacterial efficacy of clay leachates against *Escherichia coli* and methicillin-resistant *Staphylococcus aureus* (MRSA) [2]. These studies have stimulated interest in the identification of clays from other localities that possess antibacterial activity and could be utilized as new antibacterial agents.

In the food industry, studies have found a high degree of cross-contamination of various pathogens including *Salmonella typhimurium*, a Gram-negative enterobacterial pathogen and *Staphylococcus aureus*, a Gram-positive species also associated with skin infections due to inadequate cleaning and disinfection of cutting boards [3,4]. The most common disinfecting agents used to control bacterial proliferation in the food industry are the peroxygens and chlorine-based compounds. In Malaysia, *Salmonella* and *S. aureus* have been isolated from multiple food environments, but their survival against hydrated clays has not been investigated. In view of emerging science describing clay as an efficient killing agent against several bacterial pathogens, this study investigates the antibacterial activity of clay from Selangor, Malaysia against food-borne bacterial pathogens in an effort to demonstrate the potential of naturally produced clay as alternative sanitizing agents for the food industry.

Only a small proportion of clay minerals have been proven to be antibacterial [5,6,7]. In Malaysia, clay-rich soils are derived from erosion of metamorphic and sedimentary rocks, and marine alluvium deposits [8]. Locally sourced antibacterial clay has not yet been reported from this region. The search for antibacterial clays should be focused on locations where clay deposits possess characteristics similar to those identified in previous studies. Soil types with a high clay content, acidic pH [9,10] and high levels of Al^3+^ and reduced Fe^2+^ [6] were located around Selangor, Malaysia based on data compiled by Paramananthan [8].

The antibacterial activity of clays is somewhat variable, since no natural clay minerals are precisely the same due to differences in mineralogical and geochemical composition. However, the antibacterial activity of clay leachates is widely reported to be due to metal ion toxicity released from the clay mineral interlayer. Although clays have fundamental structural and chemical characteristics in common, each clay mineral has its own unique properties that determine how it will associate with other species. Cunningham et al. [9] and Williams [10] reported the importance of pH as a crucial parameter in preliminary assessment of antibacterial activity, as an acidic environment of hydrated clay minerals contributes to antibacterial efficacy by increasing the availability and toxicity of metal ions. Investigation of the antibacterial activity of natural clays against methicillin-resistant *Staphylococcus aureus* (MRSA) revealed that illite and montmorillonite perform better than kaolinite, with the high cation-exchange capacity (CEC) of clay allowing free exchange of metal ions from the surface of the particles into the surrounding medium [11]. In fact, clay minerals with high CEC have been targeted in the creation of synthetic antibacterial materials [12,13]. Known antibacterial metal ions, such Ag, Cu, Fe and Al, were commonly selected as active ingredients due to their strong inhibitory and bactericidal effects [14,15,16,17]. Previously, clay minerals with single metal ion incorporation were actively sought [18,19]. However, more recent studies suggest that antibacterial activity is due to multiple metal species working in concert, particularly in the presence of elevated iron concentrations [6,9].

This study aims to discover clays with antibacterial activity in the tropical soils in Malaysia and to examine their antibacterial activity against representative food-borne pathogens. Although different in geological setting, knowing the properties of antibacterial clays from published work helped with the screening and selection of clays with potential antibacterial activity. The outcome of this study will serve to guide future studies aimed at screening clays from soil samples for antibacterial activity and evaluating their potential as sanitizing agents in the food industry.

## 2. Results

### 2.1. Preliminary Screening of Clay Soils for Antibacterial Activity

Among the six soils, Munchong series showed the most effective antibacterial activity, as judged in a disc diffusion assay against *Salmonella typhimurium* (11.00 ± 0.71 mm), while Carey leachate showed the greatest zone of inhibition (7.63 ± 0.48 mm) against *S. aureus* (Table 1). Most of the samples were more effective against *Salmonella typhimurium* compared to *S. aureus*, as judged by the diameter of zones of inhibition. The interpretation of the diameter of the inhibition zone has been established for antibiotics, but there has been no standard for clay soils. Based on the preliminary results, the leachate samples showed moderate sensitivity (range of 7.00–11.00 mm zone). Following this, Munchong and Carey series were selected for further antibacterial testing using different treatments as described in Section 4.3.3.

Particle size analysis (Table 2) revealed that Carey has a relatively low clay content (34.62%) compared to the other five samples, which have >50% clay content. All the clay leachate samples showed acidic pH values of <5.2 (Table 3), with Carey having the lowest value (2.81 ± 0.10).

### 2.2. Antibacterial Effect of Untreated and Treated Clay Leachate and Suspension

**Figure 1:**
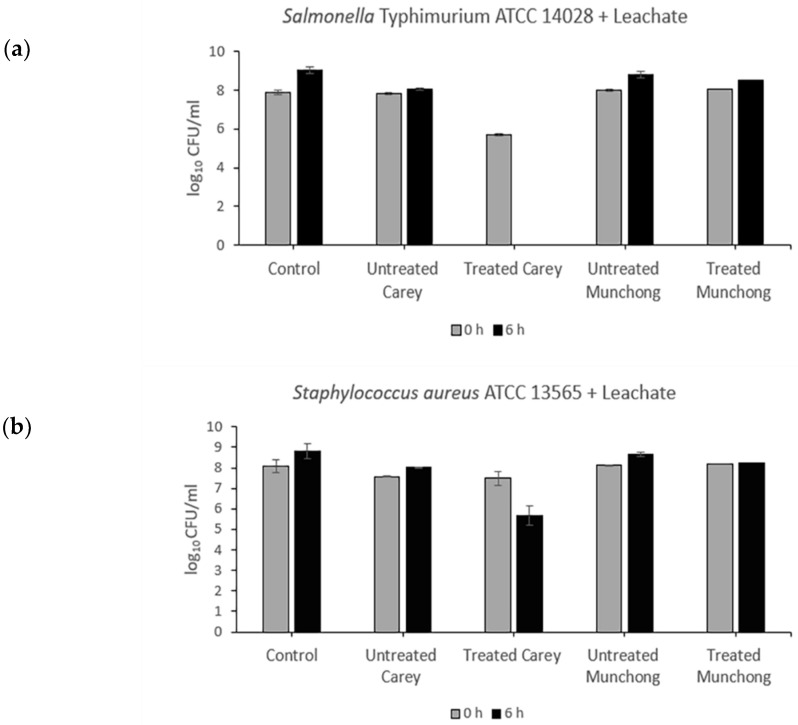
Antibacterial activity of untreated and treated clay leachates of Carey and Munchong soils following 6 h exposure to clay leachates (500 mg/mL), with sterile deionized water as control: (**a**) Viability of *Salmonella typhimurium* ATCC 14028; (**b**) Viability of *S. aureus* ATCC 13565.

**Figure 2:**
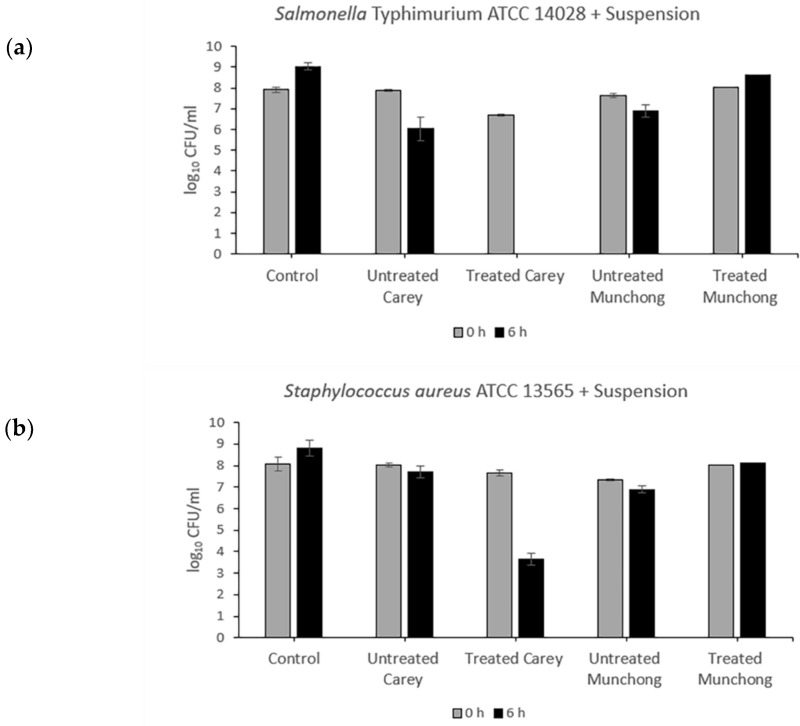
Antibacterial activity of untreated and treated clay suspensions of Carey and Munchong soils following 6 h exposure to clay leachates (500 mg/mL), with sterile deionized water as control: (**a**) Viability of *Salmonella typhimurium* ATCC 14028; (**b**) Viability of *S. aureus* ATCC 13565.

Exposure of *Salmonella typhimurium* to treated (see Section 4.2.2) Carey leachates (500 mg/mL) resulted in complete killing (5.7-log_10_ reduction), while for *S. aureus,* viability decreased by 1.82-log_10_ (Figure 1). The other three leachate samples showed no antibacterial activity against either *Salmonella typhimurium* or *S. aureus*. Meanwhile, the effect of clay suspension samples (500 mg/mL) on *Salmonella typhimurium* and *S. aureus* viability is shown in Figure 2. The treated Carey sample killed *Salmonella typhimurium* (6.7-log_10_ reduction) within 6 h and also resulted in a 4.02-log_10_ decrease in *S. aureus* viability. Suspensions of untreated Carey showed 1.82-log_10_ decrease in *Salmonella typhimurium* viability; however, the viability of *S. aureus* was minimally affected. Exposure to untreated Munchong resulted in modest reductions in *Salmonella typhimurium* and *S. aureus* viability, whereas suspensions of treated Munchong showed no growth inhibition when tested against these species. Overall, Carey leachates and suspensions originating from treated soils displayed the greatest antibacterial efficacy against both of the Gram-negative and Gram-positive bacteria compared to the untreated clay leachates and suspensions.

### 2.3. Cation-Exchange Capacity of Untreated and Treated Clays of Carey and Munchong Soils

The CEC values (Table 4) showed that treated samples yielded clay with higher CEC values relative to untreated samples. The treated Carey and Munchong had moderate ranges of CEC values (12–25 cmol(+)/kg) according to the classification of Hazelton and Murphy [21]. In contrast, CEC values were much lower for the untreated samples.

### 2.4. pH of Untreated and Treated Clays of Carey and Munchong Soils

The pH of the untreated and treated Carey samples had similarly low values (2.51 ± 0.05–2.84 ± 0.01), while there was a significant difference in Muchong clay, whereby the pH is more acidic in the untreated sample (3.48 ± 0.07–3.66 ± 0.05) compared to the treated Munchong (6.10 ± 0.03–6.19 ± 0.01) samples (Table 5).

### 2.5. Mineralogy of Untreated and Treated Clays of Carey and Munchong Soils

**Figure 3:**
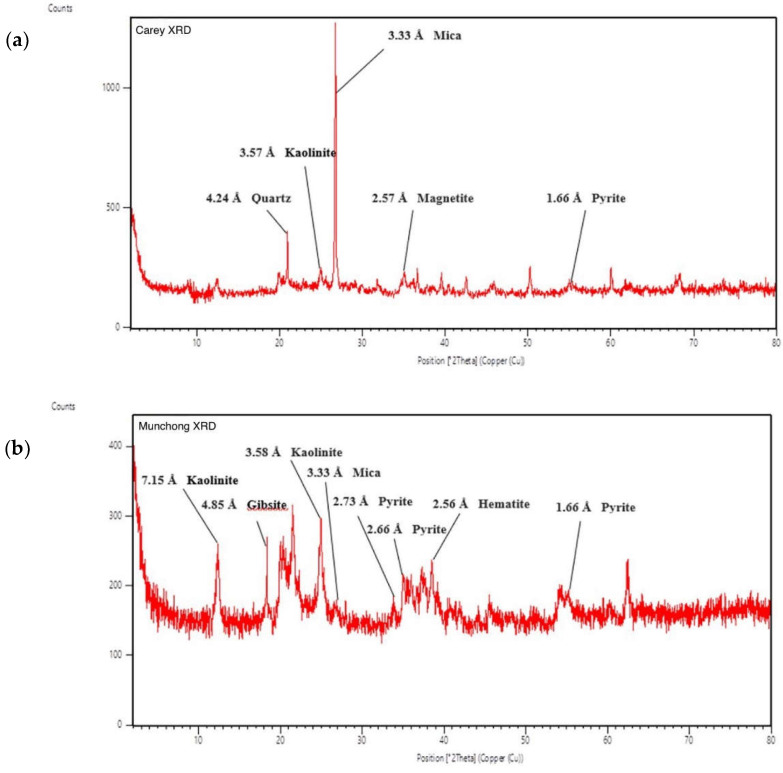
XRD (powder X-ray diffraction) graph showing peaks for mineralogy identification: (**a**) Carey clay soil; (**b**) Munchong clay soil.

The clay mineralogy of the Carey and Munchong samples revealed the presence of kaolinite, mica and pyrite in both of them (Figure 3). Kaolinite and mica as well as quartz, which are present in the Munchong sample, are common in many clay fractions [22].

The detection of pyrite and magnetite in the Carey sample indicates the presence of iron-rich mineral as part of the clay mineralogy. Meanwhile, hematite and gibbsite were present in Munchong; the primary component of each of these minerals is Fe and Al, respectively.

### 2.6. Soluble and Total Metals of Untreated and Treated Clays of Carey and Munchong Soils

Table 6 shows that, of the 10 different metals, nine metals, (the exception being Pb) are present in significantly higher levels in treated samples of Carey. Three elements, namely Al, Fe and Mg, are present at much higher concentrations in comparison to other metals. The metal analysis of Munchong samples revealed higher concentrations of Al and Fe compared to the other metals, although the values are much lower relative to the Carey samples. The Al varied greatly with a 280-fold difference between treated and untreated Munchong samples.

Total metal content analysis of Carey and Munchong revealed high Al and Fe content in both treated and untreated samples, with Mg also showing high content in the Carey samples (Table 7). The treated samples showed significant differences in all metals tested, except for Ag and Cu for Carey; for Munchong samples, the Cu, Fe, Mg and Zn content was not significantly different for both treatments.

Analysis of soluble metals in antibacterial treated Carey samples (see Table 8) revealed a higher range of concentration of Al (59.48 ± 4.05–93.63 ± 2.18 mg/L), Fe (25.64 ± 2.92–65.69 ± 3.09 mg/L) and Mg (88.48 ± 2.29–108.34 ± 2.36 mg/L). While the concentration of Al and Fe was higher in the leachates, Mg was found to be higher in the suspension samples. The concentrations of other metals were not significantly different between leachate and suspension samples.

## 3. Discussion

The aim of this study was to investigate the potential antibacterial properties of clays obtained from tropical soils. Most of the known antibacterial clays, such as the French Green and Oregon Mineral Technologies (OMT) clays, are of hydrothermal origin and contain mixed illite-smectite (expandable clay) minerals [5,6,23,24]. In searching for clays with antibacterial activity in the tropical region, six types of soil with high clay content were selected for this study. However, our preliminary results showed that high clay content in soil samples did not necessarily influence their antibacterial properties. Munchong and Batu Anam soils (>70% clay) were expected to have better potential for antibacterial activity, as clays have an important role in buffering water pH to conditions where metal ions are soluble [10]. It was also anticipated that a more acidic leachate would prove to be superior in antibacterial activity, as metal ions become more bioavailable and potentially more toxic in a low-pH solution [25]. Our preliminary disc diffusion testing showed that the acidic condition of the six clay solutions was not effective enough to inhibit the bacterial growth by itself. The antibacterial activity of Batu Anam and Bernam clay soils (pH > 5) against *Salmonella typhimurium* was not significantly different from Carey and Serkat (pH < 3) or Melaka (pH 5). Only the Munchong clay leachate with a pH 3.60 produced a significantly greater zone of inhibition (11.00 ± 0.71) when tested against the Gram-negative *Salmonella typhimurium*. In contrast to the Gram-negative bacterial screening, the Carey leachate showed the greatest activity (7.63 ± 0.48) against the Gram-positive *S. aureus*. The remaining clays with differing levels of acidity were less effective at inhibiting bacterial growth. In general, the leachates showed larger zones of inhibition with *Salmonella typhimurium* relative to *S. aureus*, indicating that Gram-negative bacteria are more susceptible. The variation found in these initial experiments may indicate that the soil preparation method and assay by disc diffusion have some limitations as means of determining the antibacterial efficacy of different types of clay samples.

Sieving served as a simple approach to collect and rapidly prepare the six soil samples. The leachates were prepared by the sieving method (untreated clay), in which larger grains of sand and silt, having particle sizes of 50–500 μm and 2–50 μm respectively, are likely to be retained. It is possible that aggregation of the clay particles and inconsistent distribution of the clay fraction could negatively affect their antibacterial activities. Particle-size fractionation is often undertaken to ensure optimal yield of the fine-sized clay mineral fraction (<2 µm) through either sedimentation or centrifugation methods. Antibacterial clays sourced from hydrothermal mineral deposits, which are mineral rich, were simply air-dried, ground and sieved before testing to prevent oxidation that could influence antibacterial activity [24]. Other studies on antibacterial clays employed the centrifugation method [26,27]. Particle size analysis revealed that Carey has a silty clay loam texture with less than 35% clay content, whereas the clay fraction was dominant (>70%) in Munchong soil. Due to the high volume of organic matter in our soil samples [8], the sedimentation approach was undertaken to recover the clay fraction, thus yielding the treated clay. The sedimentation method involves pre-treatment with hydrogen peroxide for organic matter removal and Calgon solution as a dispersing agent to produce the treated samples. However, such chemical treatment can have an effect on the dissolution of mineral constituents [28]; hence, samples were washed thoroughly with distilled water to minimise this effect.

The treated Carey clays (suspension and leachate) proved the most effective at killing *Salmonella typhimurium*. However, the viability of *S. aureus* was also reduced by the Carey clay suspension (>3 log) and leachate (<2 log), respectively. The untreated Carey and all the Munchong clays showed little or no antibacterial activity against either bacterial species. The difference in susceptibility between Gram-negative and Gram-positive bacteria has been observed with clay mineral leachates previously [9,10,26]. Otto et al. [29] reported bactericidal activity on the Gram-negative *Escherichia coli*, whereas a methicillin-resistant *S. aureus* strain was killed at a slower rate. This is likely to be due to differences in the cell-envelope architectures of these bacteria, with the outer membrane of Gram-negatives, absent from Gram-positives, representing a permeability barrier to toxic components. Caflisch et al. [30] reported that OMT clay suspensions are more effective in killing multiple strains of representative Gram-negative (*E. coli*) and Gram-positive (*S. aureus*) bacteria than clay leachates at the same concentration. While the previous study employed clay at 200 mg/mL in both suspensions and leachates, our study used a higher concentration (500 mg/mL), although the outcome is similar. These findings support a direct antibacterial contribution for clay minerals, rather than metal ion toxicity in the solution chemistry being the sole factor, as previously concluded with clay leachates [31].

We found that both untreated Carey and Munchong samples possess low CEC values, whereas the CEC values of both treated clays were higher. This fits with a higher distribution of clay in the treated sample, as the larger particles have been removed during the purification procedure for treating the clay. The increased yield of clays in the treated samples creates a larger surface area due to the finer particle size, providing enhanced clay–bacterial contacts in antibacterial assays. Although this explanation fits with the antibacterial properties of the Carey treated clays, it does not account for the reduced efficacy of the Munchong treated clays. Hence, the higher portion of clay in samples is not the sole contributor to their antibacterial properties.

The activity could be influenced by the pH of the sample, as acidic conditions should favour metal ion solubility [9]. As noted previously, pH alone is not the only factor contributing to antibacterial activity. While both *Salmonella typhimurium* and *S. aureus* are sensitive to acidic conditions [32,33], *Salmonella typhimurium* displays better capacity to survive low-pH stress (pH 3.3) [34]. However, in an acidic environment, abundant protons saturate metal binding sites in the solution, maximising the concentration of soluble metal ions. Metal ions become increasingly more bioavailable and potentially more toxic in a low-pH solution [25]. It seems likely that the abundance of metal ions in treated Carey clay samples, especially in an acidic environment, contributes to bacterial killing due to metal ion toxicity.

Carey is an acid sulfate soil, characterised by the presence of pyrite and high Al and Fe content [22]. XRD analysis confirmed the presence of pyrite, as well as magnetite in the Carey sample. The pyrite and magnetite minerals are sulfur and iron composite minerals, which could potentially contribute as sources of sulfur and iron in the clay leachate and suspension. In our study however, sulfur content was not measured. Future evaluation could be performed to analyse the potential toxicity caused by metal ions and the influence of pH on antibacterial activity.

In our effort to further determine the role of metal toxicity, we discovered that the soluble metal content was higher in treated Carey clay samples compared to the untreated ones. This finding fits with the greater antibacterial activity of treated Carey, which is also more acidic than its untreated counterpart, as judged by the bacterial log reduction value. These results are similar to previous studies which have shown that metal ion toxicity is responsible for bactericidal activity against *E. coli* and *S. aureus* (MRSA), with toxicity directly associated with the released metal ions (Fe^2+^, Cu^2+^ and Zn^2+^) in an acidic environment [2,31]. Aqueous leachates derived from known antibacterial clays tend to have high levels of Mg, Al and Ca, with some containing exceptionally high concentrations of Fe [10]. Previous work has concluded that Ca was not significant in bacterial cell death [5,35], hence we did not include analysis of Ca levels in our study. For OMT clay minerals, the dissolution of reduced Fe^2+^ and Al^3+^ was identified as the active antibacterial component, acting in concert to damage bacterial membranes [6]. Speciation modelling by Otto and Haydel [31] suggests that increasing the soluble metal ions Cu^2+^ and Fe^2+^ are the key to increasing the antibacterial activity of the leachates.

The available metals (Al, As and Cu) were higher in the treated Munchong than the untreated leachate by 280-, 80- and 4.5-fold, respectively. The higher soluble metal content could be attributed to the higher clay percentage in the treated sample, as evidenced by their larger CEC values compared to the untreated sample. However, metal analysis revealed much lower Fe, Mg, Mn, Ni, and Zn leachate content compared to both of the Carey leachate samples. This could mean that the metal content in untreated and treated Munchong is below the threshold value that could cause death of bacteria. Analysis of soluble metals in the suspension form of the antibacterial treated Carey sample revealed a significantly higher Ag and Mg content, although much lower amounts of Al, Cu and Fe in comparison to its leachate form. Both leachates and suspension killed *Salmonella typhimurium*, while the suspension displayed slightly greater antibacterial activity than the leachate when tested against *S. aureus*. Although the differences observed here were relatively small, previous studies have indicated that the continuous supply of metals in clay suspensions results in greater antibacterial efficacy compared to leachates [6].

Relative to the other three non-antibacterial leachates, the antibacterial treated Carey leachate contains significantly higher levels of available Ag, Al, Cu, Fe, Mg, Mn, Ni and Zn. Treated Carey clay has a greater CEC in comparison to the sieved sample due to increased finer clay fractions in the sample. The elevated quantities of these metals suggest that a greater amount of elements is concentrated in the finer clay fractions, as previously suggested by Williams [10], and is only released into solution upon hydration. The high total Al and Fe content in the treated Munchong clay did not seem to improve its antibacterial activity. Al is abundant as it provides the structural framework of clay minerals, but may not necessarily be present in a bioactive form. Similarly, Fe may be present in pyrite and hematite within the clay minerals but may not be readily available in a reduced state with its associated higher toxicity. On the other hand, the available metals in leachates, when present above the threshold amount, can cause death of bacteria due to metal toxicity. Williams et al. [5] reported that the leachate of antibacterial OMT clay contained significantly higher Al, Fe, Cu and Pb content, with the values of 23.9, 51.6, 0.23 and 0.00002 mg/L, respectively. These are among the metals associated with *E. coli* death. Although the metal threshold value was not determined, we can conclude, based on earlier studies, that Fe content is likely to make significant contribution to the antibacterial effectiveness of the sample against the test bacteria.

In future work, it would be helpful to determine the amount of the active antibacterial form of Fe (Fe^2+^) and how this correlates with bacterial toxicity. Further analysis of clay stability and the time course of antibacterial activity would help clarify the contribution that pH makes to metal release and the antibacterial efficacy of the Carey clay. Investigation of the mechanism of action (membrane permeability, cell surface damage and oxidative stress production) would also help to validate the clay materials as a marketable and consumable mineral-based disinfectant/sanitizing agent. In the context of Halal food assurance, the use of clays would be beneficial, not only for Islamic cleansing procedures but for effective removal of bacterial hazards along the food supply chain.

## 4. Materials and Methods

### 4.1. Soil Sampling and Preparation

**Figure A1:**
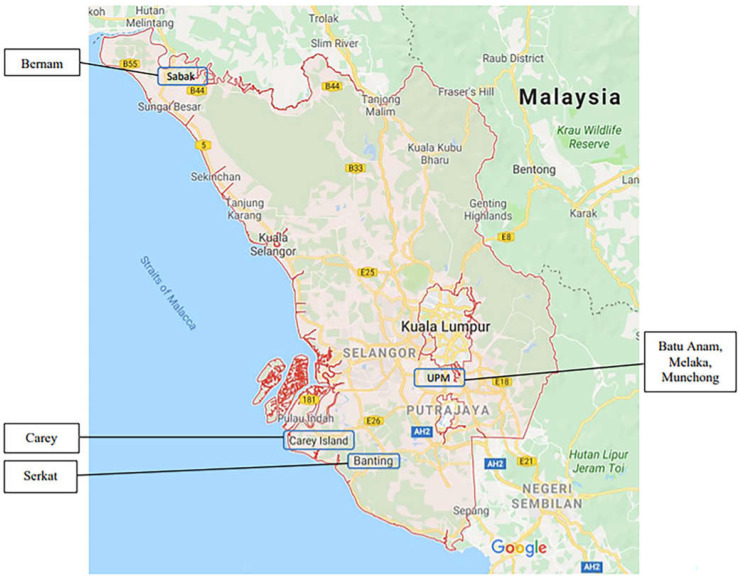
Sampling location of the six soil series.

Soil data compiled by Paramananthan [8] informed the allocation of potential sites for clay soil sampling in the state of Selangor, Malaysia. The six potential antibacterial clay soil samples, namely Batu Anam, Bernam, Carey, Melaka, Munchong and Serkat series, were collected at a depth of 25–50 cm from the surface using a hand auger, labelled and retained in polyethylene bags before transport to the laboratory for further analysis. Samples were air-dried and ground to a fine powder using a mortar and pestle. The material was passed through a 250 µm mesh sieve to collect the fine fraction. The sampling location is given in Appendix A (Figure A1).

### 4.2. Preparation of Untreated and Treated Clay Leachate and Suspension

#### 4.2.1. Untreated Clay

The fine soil sample (see Section 4.1) was further processed by passing through a 53 µm mesh sieve. This sieved soil is henceforth referred to as the untreated clay sample. Samples were autoclaved (121 °C, 20 min) to sterilize prior to experimental use.

#### 4.2.2. Treated Clay

The sieved soils (see Section 4.2.1) were further processed to separate the clay fraction from the bulk soil. The procedure involves chemical treatment and henceforth, the resulting samples are referred to as treated samples, in order to differentiate them from the untreated soil samples. The clay separation by sedimentation was performed using the method developed by Gee and Baulder [36], with minor modifications. Soil samples (250 µm), exposed to H_2_O_2_ (30%) overnight, were then heated (60 to 90 °C) to remove organic matter. Distilled water was added to a 1000 mL beaker containing the treated soil mixture and left overnight. Soil particles settled at the bottom while the upper layer of liquid was siphoned off. Calgon solution (pH 8.3) was added as a dispersing agent and the solution was stirred using a mechanical shaker (5 min) to combine both soil and water phases together. The suspension was wet-sieved using a 53 µm mesh screen to collect the finer clay-silt, while removing the coarser sand fraction. Distilled water was added to the beaker containing the silt and clay fraction and left to settle to separate the silt and clay components by sedimentation. The upper layer, containing the clay fraction, was collected by siphoning the solution into a fresh beaker and the clay was oven-dried at 105 °C to remove water. The dried fine clay was collected, gently ground in a mortar and pestle, passed through a 53 µm mesh sieve and autoclaved (121 °C, 20 min) to sterilize prior to experimental use.

#### 4.2.3. Clay Leachate

The untreated clay (see Section 4.2.1) and treated clay (see Section 4.2.2) were used to prepare untreated and treated clay leachate samples, respectively. Leachates were prepared following the method described by Borquaye et al. [37] with some modifications. Briefly, untreated or treated clays were suspended in sterile deionized water (500 mg/mL), followed by sonication (1 min), then shaken using an orbital shaker at 150 rpm for 6 h. Subsequently, the suspensions were centrifuged in an Eppendorf 5810 R using a fixed-angle rotor (4000 rpm, 4 °C, 30 min) to separate insoluble and soluble fractions. The aqueous supernatant (leachate) was recovered for experimental use.

#### 4.2.4. Clay Suspension

The untreated clay (see Section 4.2.1) and treated clay (see Section 4.2.2) were used to prepare untreated and treated clay suspension samples, respectively. A suspension was prepared by the addition of the untreated or treated clays in sterile deionized water (500 mg/mL), followed by sonication (1 min), then the mixture was shaken using an orbital shaker at 150 rpm for 6 h. The resulting suspension was employed in subsequent experiments.

### 4.3. Antibacterial Testing

#### 4.3.1. Bacterial Strains and Growth Condition

*Salmonella typhimurium* ATCC 14028, isolated from chicken tissue, and *Staphylococcus aureus* ATCC 13565, an enteroxin-producing isolate [38] from a food poisoning outbreak, were used as test organisms. Both bacterial strains were grown on nutrient agar or in nutrient broth at 37 °C for 24 h.

#### 4.3.2. Antibacterial Screening of Clay Soils

The disc diffusion method was used to screen for antibacterial activity in the six soil samples. The zone of growth inhibition was measured as described by CLSI [39]. A single bacterial colony was inoculated into 10 mL of nutrient broth (NB) and cultured for 24 h at 37 °C. The inoculum density was adjusted using NB to match the standard McFarland 0.5 (~10^8^). An aliquot of 0.1 mL of the inoculum was spread on Mueller-Hinton agar (MHA; Oxoid, Hampshire, UK). Sterile paper discs (6 mm in diameter) were immersed into each clay leachate (20 µL) for 15 min and placed onto the inoculated MHA agar plate. The plates were then incubated at 37 °C for 24 h. Bacterial growth inhibition was determined by measuring the diameter of the zones of inhibition. Sterile water was used as a negative control, while ampicillin (10 µg) and ciprofloxacin (5 µg; Fisher Scientific, Waltham, MA, USA) served as positive controls for *Salmonella typhimurium* and *S. aureus*, respectively. Testing was performed in triplicate.

#### 4.3.3. Antibacterial Effect of Untreated and Treated Clay Leachate and Suspension

The antibacterial activity of both Carey and Munchong clay leachates (see Section 4.2.3) and suspensions (see Section 4.2.4) was further tested using methods developed by Williams et al. [5] and Morrison et al. [24], with minor modifications. Bacteria were grown in nutrient broth (Oxoid, Hampshire, UK). Samples were mixed with bacteria in log-phase growth containing approximately 10^6^–10^8^ CFU/mL, in a 1:1 ratio by volume, then continuously mixed in an incubator shaker (120 rpm, 37 °C, 6 h). After 6 h of exposure, 0.1 mL of sample was removed, serially diluted in 0.1% peptone water, and plated onto nutrient agar (NA). Colonies were enumerated after incubation for 24 h at 37 °C, and results expressed as log CFU/mL.

### 4.4. Physicochemical Properties of Soil Clays

#### 4.4.1. Soil Particle Analysis

Particle size distribution was determined by the pipette method [36], using a sedimentation cylinder to determine the percentage of sand, silt and clay in each sample. The soil sample was treated with Calgon as the dispersing agent while H_2_O_2_ (30%) was used for organic matter removal.

#### 4.4.2. pH Analysis

The pH of leachate and suspension samples was measured using a Mettler Toledo Model Sevengo Pro-SG78 (Mettler Toledo International, Columbus, OH, USA) probe meter. The pH was determined in triplicate for improved accuracy.

#### 4.4.3. Cation-Exchange Capacity

Cation-exchange capacity (CEC) of clays refers to the capacity of clays to adsorb and exchange cations. The CECs of untreated and treated samples were determined by the leaching method [40]. Briefly, exchange sites of clay samples were saturated with ammonium ions (1 M NH_4_OAc, pH 7). Excess free ammonium ions (NH_4_^+^) were removed with alcohol and subsequently replaced by 1 N K_2_SO_4_. The displaced NH4^+^ was analyzed in a Perkin Elmer AAnalyst 4000 Atomic Absorption Spectrometer (AAS).

### 4.5. Mineralogical Identification

Clay mineralogy of the treated clay samples was studied using XRD-6000 (Shimadzu, Kyoto, Japan) for powder X-ray diffraction. X’Pert HighScore Plus software was used for qualitative phase analysis.

### 4.6. Metal Composition Analysis

The concentration of water-soluble metals Ag, Al, As, Cu, Fe, Mg, Mn, Ni, Pb and Zn in leachates of untreated and treated samples was determined by inductively coupled plasma optical emission spectrometry (ICP-OES; Perkin Elmer, Optima 8300). Meanwhile, the metals were extracted using the Aqua Regia method where the samples were digested with HCl and HNO_3_. The total metal concentration was determined as a reference value. These metal concentrations were determined by ICP-OES as with the leachate and suspension samples. For the suspension, the sample from Section 4.2.4 was centrifuged (4000 rpm, 4 °C, 30 min) and the resultant supernatant was collected for analysis.

### 4.7. Statistical Analysis

All analytical determinations and measurements were performed at least in triplicate, and the mean calculated by analysis of variance (ANOVA) using Minitab 18 Software. Significant differences between means were determined by the Tukey test. The level of significance for all statistical analyses was 5%.

## 5. Conclusions

This study examined the potential of soils from a tropical region to be used as antibacterial agents against selected foodborne pathogens. The leachate and suspension of the treated Carey clay samples were bactericidal against *Salmonella typhimurium*, while activity against *S. aureus* showed a significant reduction in viability. The high content of available metals, namely Ag, Al, Cu, Fe, Mg, Mn, Ni and Zn in the treated Carey clays, are the factors that are responsible for the antibacterial activity against *Salmonella typhimurium* and *S. aureus*.

## Figures and Tables

**Table 1:** Zones of inhibition of untreated clay leachates obtained from six soil series against *Salmonella typhimurium* and *S. aureus*.

Soil Series	Zone of Inhibition (mm) ^c^	
	*Salmonella typhimurium*	*S. aureus*
Batu Anam	8.82 ± 0.98 ^b^	6.36 ± 0.16 ^b^
Bernam	8.50 ± 0.58 ^b^	6.29 ± 0.15 ^b^
Carey	8.06 ± 0.43 ^b^	7.63 ± 0.48 ^a^
Melaka	7.83 ± 1.04 ^b^	6.32 ± 0.16 ^b^
Munchong	11.00 ± 0.71 ^a^	6.30 ± 0.11 ^b^
Serkat	9.12 ± 0.79 ^b^	6.26 ± 0.13 ^b^

^a,b^ Pairs of values with different lowercase superscripts indicate a significant difference (*p* < 0.05) between rows. ^c^ Positive controls with ampicillin (10 µg) against *Salmonella typhimurium* and with ciprofloxacin (5 µg) against *S. aureus* produced zones of growth inhibition of 30.69 ± 0.96 and 26.35 ± 0.67 mm, respectively.

**Table 2:** Soil particle size analysis of six soil series.

Soil Series	Soil Particle Size (%)			Texture Class ^1^
	Clay	Silt	Sand	
Batu Anam	77.84	8.51	13.96	Clay
Bernam	61.89	37.32	0.79	Clay
Carey	34.62	50.28	15.11	Silty clay loam
Melaka	52.40	7.56	39.96	Clay
Munchong	78.11	9.03	12.85	Clay
Serkat	58.08	13.41	29.73	Clay

^1^ Texture classification of the soil particle size (%) obtained from this study are referenced from the USDA manual [20].

**Table 3:** pH values of untreated clay leachates obtained from six soil series.

Soil Series	pH
Batu Anam	5.13 ± 0.11
Bernam	5.14 ± 0.02
Carey	2.81± 0.10
Melaka	4.79 ± 0.02
Munchong	3.60 ± 0.18
Serkat	2.98 ± 0.04

**Table 4:** Cation-exchange capacity of untreated and treated clay samples.

Sample	CEC (cmol(+)/kg)	Classification ^a^
Untreated Carey	9.67 ± 0.16	Low
Treated Carey	15.00 ± 0.63	Moderate
Untreated Munchong	7.11 ± 0.08	Low
Treated Munchong	21.33 ± 0.43	Moderate

^a^ Low: 6–12 cmol(+)/kg; moderate: 12–25 cmol(+)/kg [21].

**Table 5:** pH of untreated and treated clay samples.

Sample	pH	
	Leachate	Suspension
Untreated Carey	2.80 ± 0.09	2.84 ± 0.01
Treated Carey	2.51 ± 0.05	2.75 ± 0.04
Untreated Munchong	3.66 ± 0.05	3.48 ± 0.07
Treated Munchong	6.19 ± 0.01	6.10 ± 0.03

**Table 6:** Soluble metal content in untreated and treated Carey and Munchong leachates.

Metal Content (mg/L)	Carey		Munchong	
	Untreated	Treated	Untreated	Treated
Ag	0.002 ± 0.001 ^b^	0.01 ± 0.00 ^a^	0.003 ± 0.001 ^a^	<0.001 ^b^
Al	9.92 ± 2.28 ^b^	92.63 ± 2.18 ^a^	0.13 ± 0.06 ^b^	36.45 ± 1.98 ^a^
As	0.02 ± 0.01 ^b^	0.07 ± 0.01 ^a^	0.01 ± 0.00 ^b^	0.40 ± 0.03 ^a^
Cu	0.002 ± 0.001 ^b^	0.02 ± 0.00 ^a^	0.002 ± 0.000 ^b^	0.01 ± 0.00 ^a^
Fe	43.45 ± 2.67 ^b^	65.69 ± 3.09 ^a^	1.89 ± 0.22 ^a^	6.38 ± 4.03 ^a^
Mg	56.88 ± 4.65 ^b^	88.48 ± 2.29 ^a^	0.27 ± 0.00 ^a^	0.11 ± 0.03 ^b^
Mn	0.12 ± 0.02 ^b^	0.21 ± 0.02 ^a^	0.01 ± 0.00 ^a^	<0.001 ^b^
Ni	0.01 ± 0.00 ^b^	0.06 ± 0.01 ^a^	<0.001 ^a^	<0.001 ^a^
Pb	0.002 ± 0.001 ^a^	0.004 ± 0.001 ^a^	0.01 ± 0.01 ^a^	0.01 ± 0.01 ^a^
Zn	0.05 ± 0.01 ^b^	0.14 ± 0.02 ^a^	0.004 ± 0.000 ^a^	0.003 ± 0.001 ^b^

^a,b^ Pairs of values (mg/L) with different lowercase superscripts indicate a significant difference (*p* < 0.05) between groups.

**Table 7:** Total metal content in untreated and treated Carey and Munchong solid samples.

Metal Content (mg/L)	Carey		Munchong	
	Untreated	Treated	Untreated	Treated
Ag	0.32 ± 0.04 ^a^	0.48 ± 0.24 ^a^	0.04 ± 0.00 ^b^	0.18 ± 0.06 ^a^
Al	29,450 ± 678 ^b^	71,660 ± 700 ^a^	123,620 ± 17740 ^b^	213,680 ± 6280 ^a^
As	2.22 ± 0.54 ^b^	3.82 ± 0.26 ^a^	14.94 ± 3.70 ^b^	25.52 ± 1.28 ^a^
Cu	4.04 ± 0.08 ^a^	6.64 ± 5.60 ^a^	8.20 ± 0.28 ^a^	7.96 ± 0.44 ^a^
Fe	3800 ± 109 ^b^	4924 ± 16 ^a^	5382 ± 98 ^a^	5502 ± 26 ^a^
Mg	261.52 ± 1.12 ^b^	353.92 ± 4.64 ^a^	13.76 ± 0.64 ^a^	23.92 ± 9.60 ^a^
Mn	18.76 ± 1.56 ^b^	40.88 ± 0.56 ^a^	30.90 ± 0.74 ^a^	12.76 ± 0.68 ^b^
Ni	0.64 ± 0.00 ^b^	2.76 ± 0.00 ^a^	1.48 ± 0.24 ^b^	2.60 ± 0.08 ^a^
Pb	3.46 ± 0.02 ^b^	7.04 ± 0.60 ^a^	1.58 ± 0.26 ^b^	3.78 ± 0.42 ^a^
Zn	11.60 ± 2.00 ^b^	54.02 ± 1.82 ^a^	13.62 ± 1.78 ^a^	13.10 ± 1.22 ^a^

^a,b^ Pairs of values (mg/L) with different lowercase superscripts indicate a significant difference (*p* < 0.05) between groups.

**Table 8:** Soluble metal content in treated Carey samples (leachate and suspension).

Soluble Metals (mg/L)	Leachate	Suspension
Ag	0.01 ± 0.00 ^b^	0.28 ± 0.00 ^a^
Al	92.63 ± 2.18 ^a^	59.48 ± 4.05 ^b^
As	0.07 ± 0.01	n.d.
Cu	0.02 ± 0.00 ^a^	0.01 ± 0.00 ^b^
Fe	65.69 ± 3.09 ^a^	25.64 ± 2.92 ^b^
Mg	88.48 ± 2.29 ^b^	108.34 ± 2.36 ^a^
Mn	0.21 ± 0.02 ^a^	0.25 ± 0.03 ^a^
Ni	0.06 ± 0.01 ^a^	0.05 ± 0.01 ^a^
Pb	0.004 ± 0.001 ^a^	0.004 ± 0.001 ^a^
Zn	0.14 ± 0.02 ^a^	0.13 ± 0.02 ^a^

^a,b^ Pairs of values (mg/L) with different lowercase superscripts indicate a significant difference (*p* < 0.05) between groups.

## Data Availability

Not applicable.

## Sample Availability

Not available.

## References

[1] Williams L.B., Holland M., Eberl D.D., Brunet T., Brunet de Courrsou L. (2004). Killer Clays! Natural antibacterial clay minerals. Mineral. Soc. Bull..

[2] Otto C.C., Koehl J.L., Solanky D., Haydel S.E. (2014). Metal ions, not metal-catalyzed oxidative stress, cause clay leachate antibacterial activity. PLoS ONE.

[3] Redmond E.C., Griffith C.J. (2003). Consumer food handling in the home: A review of food safety studies. J. Food Prot..

[4] Di Ciccio P., Vergara A., Festino A.R., Paludi D., Zanardi E., Ghidini S., Ianieri A. (2015). Biofilm formation by *Staphylococcus aureus* on food contact surfaces: Relationship with temperature and cell surface hydrophobicity. Food Control.

[5] Williams L.B., Metge D.W., Eberl D.D., Harvey R.W., Turner A.G., Prapaipong P., Poret-Peterson A.T. (2011). What makes a natural clay antibacterial?. Environ. Sci. Technol..

[6] Morrison K.D., Misra R., Williams L.B. (2016). Unearthing the antibacterial mechanism of medicinal clay: A geochemical approach to combating antibiotic resistance. Sci. Rep..

[7] Londono S.C., Hartnett H.E., Williams L.B. (2017). Antibacterial activity of aluminum in clay from the Colombian Amazon. Environ. Sci. Technol..

[8] Paramananthan S. (2000). Soils in Malaysia: Their Characteristics and Identification.

[9] Cunningham T.M., Koehl J.L., Summers J.S., Haydel S.E. (2010). pH-dependent metal ion toxicity influences the antibacterial activity of two natural mineral mixtures. PLoS ONE.

[10] Williams L.B. (2017). Geomimicry: Harnessing the antibacterial action of clays. Clay Miner..

[11] Otto C.C., Kilbourne J., Haydel S.E. (2016). Natural and ion-exchanged illite clays reduce bacterial burden and inflammation in cutaneous meticillin-resistant *Staphylococcus aureus* infections in mice. J. Med. Microbiol..

[12] Bagchi B., Kar S., Kr S., Bhandary S., Roy D., Kr T., Das S., Nandy P. (2013). In situ synthesis and antibacterial activity of copper nanoparticle loaded natural montmorillonite clay based on contact inhibition and ion release. Colloids Surf. B Biointerfaces.

[13] Jiang J., Zhang C., Zeng G.M., Gong J.L., Chang Y.N., Song B., Deng C.H., Liu H.Y. (2016). The disinfection performance and mechanisms of Ag/lysozyme nanoparticles supported with montmorillonite clay. J. Hazard. Mater..

[14] Berger T.J., Spadaro J.A., Chapin S.E., Becker R. (1976). Electrically generated silver ions: Quantitative effects on bacterial and mammalian cells. Antimicrob Agents Chemother..

[15] Domek M.J., Lechevallier M.W., Cameron S.C., Mcfeters G.A. (1984). Evidence for the role of copper in the injury process of coliform bacteria in drinking water. Appl. Environ. Microbiol..

[16] Gordon A.S., Howell L.D., Harwood V. (1994). Responses of diverse heterotrophic bacteria to elevated copper concentrations. Can. J. Microbiol..

[17] Nies D.H. (1999). Microbial heavy-metal resistance. Appl. Microbiol. Biotechnol..

[18] Hu C.H., Xia M.S. (2006). Adsorption and antibacterial effect of copper-exchanged montmorillonite on *Escherichia coli* K88. Appl. Clay Sci..

[19] Magaña S.M., Quintana P., Aguilar D.H., Toledo J.A., Ángeles-Chávez C., Cortés M.A., León L., Freile-Pelegrín Y., López T., Sánchez R.M.T. (2008). Antibacterial activity of montmorillonites modified with silver. J. Mol. Catal. A Chem..

[20] USDA (1987). Soil Mechanics Level 1. Module 3-USDA Textural Soil Classification Study Guide.

[21] Hazelton P., Murphy B. (2007). Interpreting Soil Test Results: What do All the Numbers Mean?.

[22] Shamshuddin J. (2006). Acid Sulfate Soils in Malaysia.

[23] Williams L.B., Haydel S.E., Giese R.F., Eberl D.D. (2008). Chemical and mineralogical characteristics of French green clays used for healing. Clays Clay Min..

[24] Morrison K.D., Underwood J.C., Metge D.W., Eberl D.D., Williams L.B. (2013). Mineralogical variables that control the antibacterial effectiveness of a natural clay deposit. Environ. Geochem. Health.

[25] Wang L.K., Hung Y.T., Shammas N.K. (2009). Handbook of Advanced Industrial and Hazardous Wastes Treatment.

[26] Haydel S.E., Remenih C.M., Williams L.B. (2008). Broad-spectrum in vitro antibacterial activities of clay minerals against antibiotic-susceptible and antibiotic-resistant bacterial pathogens. J. Antimicrob. Chemother..

[27] Ibbini J.H., Al-qinna M.I., Mashal K.Y., Abuidhail J., Alzoubi K.H., Masadeh M.M. (2018). Are clay minerals in Jordanian soils antibacterial?. Jordan J. Earth Environ. Sci..

[28] Jensen J.L., Schj P., Watts C.W., Christensen B.T., Munkholm L.J. (2017). Soil texture analysis revisited: Removal of organic matter matters more than ever. PLoS ONE.

[29] Otto C.C., Cunningham T.M., Hansen M.R., Haydel S.E. (2010). Effects of antibacterial mineral leachates on the cellular ultrastructure, morphology, and membrane integrity of *Escherichia coli* and methicillin-resistant *Staphylococcus aureus*. Ann. Clin. Microbiol. Antimicrob..

[30] Caflisch K.M., Schmidt-Malan S.M., Mandrekar J.N., Karau M.J., Nicklas J.P., Williams L.B., Patel R. (2018). Antibacterial activity of reduced iron clay against pathogenic bacteria. Int. J. Antimicrob. Agents.

[31] Otto C.C., Haydel S.E. (2013). Exchangeable ions are responsible for the in vitro antibacterial properties of natural clay mixtures. PLoS ONE.

[32] Lund B., Baird-Parker A.C., Baird-Parker T.C., Gould G.W., Gould G.W. (2000). Microbiological Safety and Quality of Food.

[33] Jay J.M., Loessner M.J., Golden D.A. (2008). Modern Food Microbiology.

[34] Foster J.W. (1993). The acid tolerance response of *Salmonella typhimurium* involves transient synthesis of key acid shock proteins. J. Bacteriol..

[35] Borrok D., Fein J.B., Tischler M., Loughlin E.O., Meyer H., Liss M. (2004). The effect of acidic solutions and growth conditions on the adsorptive properties of bacterial surfaces. Chem. Geol..

[36] Gee G.W., Baulder J.W. (1986). Particle Size Analysis. Method of Soil Analysis.

[37] Borquaye L.S., Ocansey E., Semenya J. (2016). Inhibitory effect of selected Ghanaian clay leachates on some pathogenic microbes. Am. J. Microbiol. Immunol..

[38] Casman E.P., Bergdoll M.S., Robinson J. (1963). Designation of Staphylococcal enterotoxins. J. Bacteriol..

[39] CLSI (2016). Performance standards for antimicrobial susceptibility testing. Clin. Lab Stand. Institute..

[40] Page A.L. (1982). Methods of Soil Analysis. Part 2. Chemical and Microbiological Properties.

